# Increased fructose consumption has sex‐specific effects on fibroblast growth factor 21 levels in humans

**DOI:** 10.1002/osp4.360

**Published:** 2019-08-30

**Authors:** M. Rodgers, B. Heineman, J. Dushay

**Affiliations:** ^1^ Division of Endocrinology and Metabolism Beth Israel Deaconess Medical Center Boston MA USA

**Keywords:** FGF21, fructose, metabolism, OFTT

## Abstract

**Objective:**

Fibroblast growth factor 21 (FGF21), a primarily hepatic hormone with pleotropic metabolic effects, is regulated by fructose in humans. Recent work has established that 75 g of oral fructose robustly stimulates FGF21 levels in humans with peak levels occurring 2 h following ingestion; this has been termed an oral fructose tolerance test (OFTT). It is unknown whether prolonged high‐fructose consumption influences the FGF21 response to acute fructose or whether biological sex influences FGF21–fructose dynamics.

**Methods:**

Thirty‐nine healthy adults underwent baseline OFTT following an overnight fast. For the high‐fructose exposure protocol, 20 subjects ingested 75 g of fructose daily for 14 ± 3 d, followed by repeat OFTT. For the control group, an OFTT was repeated following 14 ± 3 d of *ad lib* diet. For all subjects, FGF21 levels, glucose, insulin, non‐esterified fatty acids and triglyceride levels were measured at baseline and 2 h following OFTT. All subjects maintained 3‐d food logs prior to OFTT testing.

**Results:**

Women demonstrated significantly higher baseline and peak stimulated total and intact FGF21 levels compared with men both before and after high‐fructose exposure. Baseline total and intact FGF21 levels decreased following ongoing fructose exposure, maintaining a stable ratio. This decrease was sex specific, with only women demonstrating decreased baseline FGF21 levels. There were no changes in metabolic or anthropometric parameters following the high‐fructose exposure.

**Conclusions:**

Daily ingestion of 75 g of fructose for 2 weeks results in a sex‐specific decrease in baseline FGF21 levels without change in body weight or biochemical evidence of metabolic injury. There were also sex‐specific differences in peak fructose‐stimulated FGF21 levels, which do not change with high‐fructose consumption. The role of FGF21 in the development of metabolic disease caused by fructose consumption may differ based on biological sex. Future long‐term studies should consider sex differences in FGF21–fructose dynamics.

## 1 Introduction

Fibroblast growth factor 21 (FGF21) is a hormone made primarily in the liver and an important regulator of metabolism in both rodents and humans. In rodent models of obesity and monkeys with diabetes, exogenous FGF21 improves glucose tolerance 1, 2, and FGF21 analogues are in clinical development for the treatment of several metabolic diseases in humans 3. Conversely, mice deficient in FGF21 have obesity and cannot metabolize lipids when given a ketogenic diet 4, 5.

Diet composition has been shown by many groups to influence FGF21 levels in rodents; however, fewer studies have explored the effects of dietary manipulation on FGF21 levels in humans. Human consumption of fructose has been linked to the development of metabolic disease, including obesity, insulin resistance and non‐alcoholic fatty liver disease 6, 7. In particular, high‐fructose corn syrup has been implicated as a contributor to the growing epidemic of obesity and a major factor in the overall increase in fructose consumption in the USA 8. In humans, FGF21 levels respond acutely to an oral fructose load, with peak levels occurring 2 h following ingestion of a pure fructose beverage 9. There is also an FGF21 dose–response to acute fructose consumption, with higher doses of fructose resulting in higher peak FGF21 levels 10.

Most cross‐sectional human studies have shown that individuals with obesity have higher FGF21 levels compared with lean counterparts 7, 11, 12, 13, 14, 15. Less is known about factors other than metabolic health that influence FGF21 levels, such as biological sex. In rats, a high‐fat, high‐fructose diet results in sex‐specific differences in hepatic steatosis, inflammation and FGF21 expression 16. In humans, there are conflicting data regarding the association between biological sex, FGF21 levels and metabolic consequences of fructose consumption. A Danish population study found that girls have significantly higher fasting FGF21 levels compared with boys 17; however, other studies in lean humans have not found differences in circulating FGF21 levels between men and women 7, 18, and not all population studies have been stratified by sex. With respect to fructose consumption, one study found that healthy women fed a high‐fructose meal demonstrated a greater increase in postprandial hepatic *de novo* lipogenesis (DNL) compared with men, 19 while another group reported that men had greater fructose‐induced stimulation of DNL compared with women 20.

Given the widespread consumption of fructose in popular sugar‐sweetened beverages, it is of great clinical and public health interest to understand the metabolic impact of chronic fructose exposure. To date, no studies have examined the effects of ongoing, high‐dose fructose intake on FGF21 dynamics in humans or whether there are sex‐specific differences in the FGF21 response to persistent high‐fructose consumption. The study herein hypothesizes that ongoing, high‐fructose consumption will lead to increased baseline and FGF21 levels, reflecting stress to the liver and that there may be sex‐based differences in FGF21–fructose dynamics following a period of high‐fructose consumption.

## 2 Materials and methods

### 2.1 Subjects

Lean, healthy male and female subjects were recruited through online advertisements. Inclusion criteria were body mass index (BMI) 19–25 kg m^−2^, age 18–60, no chronic medical conditions and no chronic medication use other than oral contraceptive pills (OCPs) or thyroid hormone (no change in dose for 6 months, with thyroid‐stimulating hormone in the normal range at the screening visit). Exclusion criteria included impaired fasting glucose, diabetes, hyperlipidaemia, hypertension, liver disease, kidney disease, recent weight gain or loss or history of fructose intolerance. All subjects provided written informed consent. A screening visit included a review of medical history, physical exam and baseline laboratory tests. All visits were conducted at the Harvard Catalyst Clinical Research Center at the Beth Israel Deaconess Medical Center (BIDMC) in Boston, Massachusetts, in accord with the Declaration of Helsinki. The research protocols were approved by the BIDMC Institutional Review Board (ClinicalTrials.gov identifiers: NCT03201549 and NCT02884791).

### 2.2 Study protocol

Thirty‐nine subjects completed two study visits at the Harvard Catalyst BIDMC Center Clinical Research Center. Nine men and 11 women were enrolled in the intervention group and nine men and 10 women in the control group. At both study visits, subjects drank a beverage containing 75 g of pure fructose dissolved in 225 ml of water (oral fructose tolerance test [OFTT]) following an overnight fast of at least 8 h. Blood was drawn before consumption of the solution and 2 h following for measurement of glucose, insulin, triglycerides, non‐esterified fatty acids (NEFA) and liver enzymes. Starting the following day, participants in the high‐fructose intervention group (*n* = 20) ingested 75 g of pure fructose powder daily for 14 ± 3 d. The fructose was consumed according to subjects' individual preferences, including being mixed into beverages or solid food as a single dose or as multiple small doses over 24 h. Subjects confirmed consumption of the full dose of fructose on a daily basis via text message or email to the study team. A second OFTT was performed immediately following the final day of high‐fructose consumption, again after an overnight fast. Subjects completed 3‐d food records prior to each OFTT. Subjects in the control group (*n* = 19) followed all of the above study procedures but consumed their usual *ad lib* diet for 14 ± 3 d between the two 75 g of OFTTs.

### 2.3 Biochemical analysis

Total serum FGF21 levels were measured using the Human FGF21 Quantikine ELISA kit from R&D Systems. Intact serum FGF21 levels were measured using the Human Intact FGF21 ELISA kit from Eagle Biosciences. Serum NEFAs were measured using the HR series NEFA assay from Wako Diagnostics. Serum insulin was measured using the Human Insulin ELISA kit from Crystal Chem. Measurements of glucose, total cholesterol, high‐density lipoprotein, low‐density lipoprotein, triglycerides, alanine aminotransferase and aspartate aminotransferase were performed at LabCorp using standard procedures.

### 2.4 Calculations and statistical analyses

All comparisons of the pre‐intervention and post‐intervention parameters were performed with paired samples *t*‐tests. Comparisons of the control and intervention groups and of men and women were performed with the Student's *t*‐test.

## 3 Results

### 3.1 Fibroblast growth factor 21 response to high‐fructose consumption

**Figure 1:**
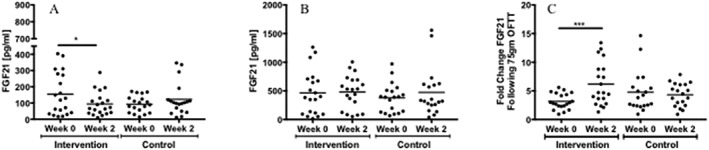
(A) Fasting fibroblast growth factor 21 (FGF21) levels before (week 0) and after (week 2) the study period for both groups. There was a significant decrease in fasting FGF21 following 2 weeks of high‐fructose consumption (^*^ p < 0.05). (B) Fructose‐stimulated FGF21 levels before (week 0) and after (week 2) the study period for both groups. (C) Fold change of FGF21 following 75 g of oral fructose tolerance test (OFTT) for both groups. There was a significant increase in the fold change of FGF21 for the intervention group following the study period (^***^ p < 0.0005).

There were no significant differences in baseline characteristics (age, weight, BMI, fasting glucose and fasting lipids) between the high‐fructose intervention and control groups. The mean age of our study population was 22 years, mean BMI was 22 kg m^−2^ and mean fasting glucose was 82 mg dl^−1^. Intervention and control groups showed no differences in average daily *ad lib* fructose consumption at baseline (intervention group: 18.6 ± 5.3 g d^−1^ and control group: 10.3 ± 1.5 g d^−1^, *p* = 0.24) or during the last 3 d of the study period (intervention group: 17.5 ± 6.5 g d^−1^ and control group: 13.8 ± 3.5 g d^−1^, *p* = 0.67). There were no changes in weight, BMI, alanine aminotransferase, aspartate aminotransferase, glucose, insulin, homeostatic model assessment–insulin resistance score, triglycerides or NEFA following the 2‐week study period in either group (Table 1). Baseline total and intact FGF21 levels decreased significantly after 2 weeks of fructose exposure in the intervention group (70.6 ± 17.3 to 40.4 ± 10.0 pg ml^−1^, *p* = 0.03) but not in the control group (66.7 ± 11.6 to 87.6 ± 16.7 pg ml^−1^, *p* = 0.22) corresponding to a significant increase in fold change of FGF21 following OFTT (Table 1 and Figure 1A,C). OFTT‐stimulated peak total FGF21 values did not change after the study period for either group as a whole (intervention group: baseline 465.1 ± 86.1 pg ml^−1^ and post‐intervention 481.1 ± 65.1 pg ml^−1^, *p* = 0.83; control group: baseline 378.1 ± 58.2 pg ml^−1^ and post‐intervention 472.3 ± 95.0 pg ml^−1^, *p* = 0.11) (Table 1 and Figure 1B). There was similarly no change in peak stimulated intact FGF21 levels between the intervention and control groups (Table 1).

**Table 1:** Metabolic parameters for the intervention (n = 20) and control (n = 19) groups before (week 0) and after (week 2) the study period

	Intervention			Control		
	Week 0	Week 2	p	Week 0	Week 2	p
Weight (kg)	63.95 ± 2.11	63.92 ± 2.13	0.87	65.24 ± 1.81	64.81 ± 1.77	0.24
BMI (kg m^−2^)	22.16 ± 0.46	22.20 ± 0.45	0.57	22.90 ± 0.34	22.73 ± 0.33	0.22
ALT (IU L^−1^)	15.75 ± 1.42	14.35 ± 1.18	0.13	22.74 ± 4.93	14.00 ± 1.39	0.09
AST (IU L^−1^)	22.30 ± 1.62	20.65 ± 1.32	0.29	19.21 ± 1.37	19.05 ± 1.31	0.90
Fasting glucose (mg dl^−1^)	82.30 ± 1.76	81.45 ± 1.06	0.54	83.37 ± 1.53	82.58 ± 1.81	0.58
Fasting insulin (mU L^−1^)	9.50 ± 1.38	7.33 ± 1.20	0.22	5.61 ± 0.39	5.80 ± 0.64	0.71
HOMA‐IR score	1.96 ± 0.30	1.47 ± 0.24	0.21	1.15 ± 0.08	1.19 ± 0.13	0.72
Fasting triglycerides (mg dl^−1^)	79.80 ± 8.64	80.80 ± 8.39	0.90	75.21 ± 6.54	69.05 ± 6.04	0.35
Fasting NEFA (mEq L^−1^)	51.90 ± 6.16	60.16 ± 10.15	0.49	59.59 ± 11.38	50.14 ± 5.60	0.36
Fasting total FGF21 (pg ml^−1^)	154.82 ± 29.21	94.19 ± 16.22	0.02*	92.93 ± 11.71	122.98 ± 22.81	0.17
Stimulated total FGF21 (pg ml^−1^)	465.10 ± 86.14	481.09 ± 65.10	0.83	378.06 ± 58.18	472.89 ± 95.01	0.11
Fold change of total FGF21	3.17 ± 0.31	6.19 ± 0.84	0.0003*	4.77 ± 0.82	4.32 ± 0.46	0.60
Fasting intact FGF21 (pg ml^−1^)	70.63 ± 17.37	40.38 ± 10.00	0.03*	66.69 ± 11.64	87.57 ± 16.72	0.22
Stimulated intact FGF21 (pg ml^−1^)	162.54 ± 40.41	227.68 ± 35.48	0.12	219.89 ± 40.65	256.19 ± 51.18	0.36
Fold change of intact FGF21 (pg ml^−1^)	2.51 ± 0.59	5.72 ± 1.41	0.02*	3.61 ± 0.54	3.49 ± 0.54	0.87
Baseline ratio intact: total	0.27 ± 0.04	0.25 ± 0.05	0.59	0.49 ± 0.07	0.53 ± 0.09	0.58
Stimulated ratio intact: total	0.32 ± 0.05	0.43 ± 0.05	0.053	0.54 ± 0.08	0.45 ± 0.07	0.54

Values are average ± standard error. *p* < 0.05 is considered significant.

ALT, alanine aminotransferase; AST, aspartate aminotransferase; BMI, body mass index; FGF21, fibroblast growth factor 21; HOMA‐IR, homeostatic model assessment–insulin resistance; NEFA, non‐esterified fatty acid.

* = *p* < 0.05

### 3.2 Sex differences in baseline fibroblast growth factor 21 levels and the response to high‐fructose consumption

**Figure 2:**
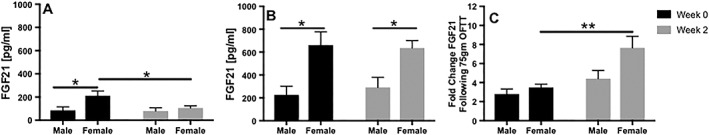
(A) Baseline fasting fibroblast growth factor 21 (FGF21) levels before and after the study period for men and women. Women had significantly higher baseline FGF21 levels and decreased baseline FGF21 levels after 2 weeks of fructose exposure (p = 0.04). (B) Fructose‐stimulated FGF21 levels before and after the study period for men and women. Women had significantly higher FGF21 levels than men at both time points (^*^ p < 0.008). (C) The fold change of FGF21 following 75 g of oral fructose tolerance test (OFTT) was significantly elevated in women after fructose intervention (^*^ p < 0.05; ^**^ p < 0.01).

Prior to high‐fructose exposure, women had significantly higher baseline and stimulated FGF21 levels compared with men (Table 2 and Figure 2A,B). Women but not men demonstrated a significant decrease in baseline total and intact FGF21 levels following ongoing fructose exposure, which corresponded to significant increases in the fold change of FGF21 following a 75 g of OFTT (Table 2 and Figure 2A,B). There were no significant differences in total or intact FGF21 levels between women taking OCPs and those not taking OCPs in either the intervention or control group for any time points (data not shown).

**Table 2:** Sex differences in the effects of high‐fructose exposure (men: n = 8; women: n = 11)

	Week 0			Week 2			Within sex	
	Male	Female	p	Male	Female	p	p (male)	p (female)
Fasting total FGF21 (pg ml^−1^)	85.70 ± 29.43	211.37 ± 40.86	0.03*	79.17 ± 28.98	106.48 ± 18.04	0.42	0.39	0.02*
Stimulated total FGF21 (pg ml^−1^)	225.26 ± 75.76	661.33 ± 115.41	0.008*	291.05 ± 88.15	636.57 ± 64.66	0.005*	0.24	0.86
Fold change of total FGF21	2.79 ± 0.53	3.48 ± 0.35	0.28	4.41 ± 0.86	7.64 ± 1.22	0.05	0.10	0.001*
Fasting intact FGF21 (pg ml^−1^)	40.57 ± 22.54	83.98 ± 22.28	0.27	36.09 ± 17.71	37.74 ± 10.05	0.93	0.72	0.03*
Stimulated intact FGF21 (pg ml^−1^)	53.52 ± 25.26	227.95 ± 53.61	0.03*	143.21 ± 65.12	278.36 ± 34.39	0.06	0.08	0.42
Fold change of intact FGF21 (pg ml^−1^)	0.84 ± 0.68	4.62 ± 1.19	0.19	2.83 ± 0.95	6.65 ± 1.65	0.14	0.08	0.04*
Baseline ratio intact: total FGF21	0.22 ± 0.09	0.33 ± 0.06	0.35	0.28 ± 0.08	0.28 ± 0.08	0.94	0.75	0.50
Stimulated ratio intact: total FGF21	0.28 ± 0.11	0.35 ± 0.06	0.55	0.38 ± 0.08	0.46 ± 0.07	0.50	0.21	0.16

Values are average ± standard error. *p* values <0.05 are considered significant.

FGF21, fibroblast growth factor 21.

* = *p* < 0.05

### 3.3 Intact and total fibroblast growth factor 21 levels following high‐fructose consumption

There was no significant change in the ratio of intact‐to‐total FGF21 levels at baseline; however, there was a trend towards an increased stimulated intact‐to‐total ratio following the high‐fructose consumption period (Table 1). There were no sex‐based differences in the intact‐to‐total FGF21 ratio following high‐fructose consumption (Table 2).

## 4 Discussion

The present study of lean, healthy men and women reports sex‐based differences in baseline FGF21 levels and in the FGF21 response to ongoing high‐fructose consumption. Before a 2‐week high‐fructose intervention, women had significantly higher fasting and OFTT‐stimulated total FGF21 levels compared with men. Additionally, women showed higher peak stimulated FGF21 levels following the high‐fructose consumption. Women demonstrated a significant decrease in baseline total and active FGF21 levels following the high‐fructose study period, which explains the sex‐specific increase in fold change of both total and active FGF21. Men did not show a significant change in total or intact FGF21 levels at any time point following the high‐fructose consumption period.

To date, literature on the physiology and regulation of FGF21 in humans indicates that this important regulatory protein acts as both a marker of metabolic disease and a hormone with anti‐inflammatory and protective effects against the development of metabolic disease. Several population studies have shown that individuals with obesity, type 2 diabetes and metabolic syndrome have higher circulating FGF21 levels 7, 11, 12, 13, 14, 15, suggesting a state of FGF21 resistance similar to insulin and leptin resistance; this physiological state would therefore be associated with increased risk for the development or worsening of dysmetabolism. Further complicating the study of FGF21 physiology in humans is the wide variability of FGF21 levels within and between individuals, with the precise biological factors contributing to the variability of this hormone remaining unknown 9, 10, 18.

In the present study, an ethnically diverse group of lean men and women without metabolic disease consumed 75 g of fructose daily over a 2‐week period while control subjects followed their usual diet. The FGF21 response to an acute fructose load was studied before and after the study period. A 14‐d intervention period was chosen in an effort to maximize compliance and because it has been demonstrated that consumption of beverages containing high‐fructose corn syrup for this duration is sufficient to confer an increase in risk factors associated with cardiovascular disease 6. Consuming 75 g of pure fructose is equivalent to consuming approximately 32 oz of a typical sugar‐sweetened carbonated beverage daily. This amount of fructose is an excessive but not unrealistic amount of additional fructose for subjects to consume daily for 2 weeks. This dose of fructose was approximately triple what subjects consumed *ad lib* in an average day based on food logs collected before and for the last 3 d of the high‐fructose intervention. Importantly, 75 g of daily fructose consumption did not cause weight gain or dysmetabolism, allowing investigation of the effect of increased fructose specifically.

Healthy humans fed a high‐carbohydrate diet for 3 d had an eightfold increase in FGF21 compared with a control group 21, raising the question of whether increased FGF21 levels are a marker of poor carbohydrate tolerance and risk for the development of metabolic disease or rather compensatory and protective. Binge ethanol consumption has also been shown to increase FGF21 levels in humans much more robustly than fructose, although with a different time course following ethanol consumption, and mice fed with alcohol both acutely and chronically have increased baseline and peak FGF21 levels 22, 23. FGF21–ethanol dynamics are thought to confer protection against the development of alcoholic liver disease 22, 23; however, the effect of chronic alcohol consumption on FGF21 levels in humans has not been reported. As well, FGF21 regulates taste preference for sugar and alcohol 23, 24, 25, and individuals with a preference for sweet foods have shown to have higher baseline FGF21 levels 26.

The present finding of decreased baseline FGF21 levels in women following prolonged high‐fructose consumption is surprising. This change may indicate increased risk for development of metabolic disease or liver injury in women, or it could be the case that decreased baseline FGF21 levels down‐regulate sweet preference in an effort to prevent ongoing excessive fructose consumption and metabolic sequelae. Taste preference was not formally studied during the high‐fructose consumption period; however, *ad lib* fructose intake did not change in the intervention group during the high‐fructose feeding period, which indirectly demonstrates that sweet preference did not change. Study participants were advised to limit alcohol consumption to no more than one serving of alcohol per day while participating in the study (most subjects abstained completely), so taste preference for alcohol could not be assessed in the present study. Future studies should formally investigate whether chronic high‐fructose intake affects taste preference for either sweet or alcohol.

Fibroblast growth factor 21 circulates in both uncleaved and cleaved forms. Fibroblast‐activating protein (FAP) cleaves and thereby inactivates FGF21 27. Measurement of total FGF21 level includes both the cleaved and uncleaved forms of the hormone, while the intact level includes only the uncleaved, active form. The ratio of intact‐to‐total FGF21 remains unchanged over the course of a 75 g of OFTT 10, but it is not known whether the ratio of intact‐to‐total FGF21 changes in response to ongoing high‐fructose exposure. The present study demonstrates that the baseline ratio of intact‐to‐total FGF21 remains stable after 2 weeks of high‐fructose consumption, while the stimulated ratio trends towards an increase; larger studies are required to determine whether this change is significant. There were no sex‐based differences in either baseline or stimulated intact‐to‐total FGF21 ratios following a period of high‐fructose consumption.

The observed stability of this ratio is consistent with the finding that the intact‐to‐total FGF21 ratio did not change in patients with acute pancreatitis, even though these individuals had significantly higher circulating levels compared with controls 28. It therefore appears that higher FGF21 levels are associated with higher cleavage of FGF21, thereby maintaining the same intact‐to‐total ratio, although neither of these studies included measurement of FAP levels. Individuals with metabolic disease may have higher circulating FGF21 levels but may also have higher levels of FAP and therefore less intact circulating FGF21, which may in turn contribute to dysmetabolism. In obese mice, pharmacological inhibition of FAP increased FGF21 levels, reduced body weight and improved glucose tolerance 29. Investigation of FAP expression in humans, as well as the ratio of intact‐to‐total FGF21 in different metabolic and inflammatory diseases, may help further elucidate if and how FGF21 exerts anti‐inflammatory or protective metabolic effects.

There are reported, albeit conflicting, sex‐based differences in DNL in response to fructose. Tran *et al*. reported that women may have lower DNL and less suppression of lipid oxidation compared with men, suggesting that women are protected against fructose‐induced hypertriglyceridaemia 20, while Low *et al*. found that consumption of a high‐fructose meal increases postprandial DNL more significantly in women compared with men 19.

Because circulating FGF21 levels correlate with DNL 30, higher baseline FGF21 levels among women in the present study is in agreement with the observation that women have higher postprandial DNL. Carbohydrate‐responsive element‐binding protein (ChREBP), which regulates both FGF21 and DNL 30, may play a role in these observed sex differences. Additionally, the gut microbiome may influence energy metabolism in part by the regulation of ChREBP 31. Given potential sex‐based differences in gut microbiota 32, men and women may regulate ChREBP and, ultimately, FGF21, differently.

The finding that women have higher baseline circulating FGF21 levels than men is also in agreement with a recent population study showing that girls have higher fasting FGF21 levels compared with boys 17 and suggests that sex‐related FGF21 differences continue from adolescence into adulthood. It is not known whether or how these differences in baseline FGF21 levels affect risk for the development of metabolic disease later in life, with or without dietary manipulation. Age itself may also have a biological effect on FGF21 levels in humans, as one study found that FGF21 increases with age in non‐obese individuals, independent of body composition 33; our own observations in a small number of older adults confirm this finding (unpublished data). In the present study, the subject population had a narrow range of age and BMI, which reduces the likelihood that physiological confounders, specifically age and adiposity, account for the sex‐related differences in FGF21 that were observed. The interactions between age, metabolic parameters and FGF21 are all important areas of future research

In order to determine whether OCPs contributed to sex‐specific differences, FGF21 levels of women taking OCPs were compared with levels among women not taking OCPs at each time point. There were no differences (data not shown) indicating that OCPs are not responsible for the changes in FGF21 levels among women. Phase of the menstrual cycle also had no effect on baseline or stimulated FGF21 levels in female subjects. Interestingly, however, oestrogen may play a role in sex‐based differences in the development of metabolic disease, particularly hepatic steatosis. Ovariectomized female rats had reduced hepatic expression of FGF21, increased hepatic steatosis and a greater accumulation of liver fat compared with non‐ovariectomized female rats, and oestrogen replacement was associated with reduced hepatic steatosis and increased FGF21 expression 16. Furthermore, fructose‐fed female rats did not develop hypertension or hyperinsulinaemia like their male counterparts unless they are ovariectomized 34. While the mechanism is unclear, female sex hormones may have a protective role in metabolism. It is important for future studies to further explore the relationship between female sex hormones and regulation of FGF21 by fructose.

This study has several limitations. The small sample size of healthy, lean young adults may reduce the generalizability of our findings. Variability of FGF21 levels within and between subjects, acknowledged by all researchers studying FGF21 physiology in humans, may affect the reproducibility of our findings. Additionally, the high‐fructose intervention period in this study was short. It is unknown whether the observed changes in FGF21 following 2 weeks of high‐fructose consumption are sustained over months or years or whether these changes are predictive of or protective against the development of metabolic disease. A much longer intervention period would be better for understanding prolonged metabolic consequences of increased fructose consumption, as the habitual consumption of foods and beverages with high‐fructose content may alter FGF21–fructose dynamics over decades.

## 5 Conclusions

Women have higher baseline FGF21 levels and peak fructose‐stimulated FGF21 levels compared with men, which persists after ongoing high‐dose fructose consumption. Daily ingestion of 75 g of fructose for 2 weeks results in a significant, sex‐specific decrease in baseline FGF21 levels, without change in body weight or biochemical evidence of hepatic or metabolic injury. The ratio of intact‐to‐total FGF21 is not affected by ongoing high‐fructose consumption in healthy young adults. These findings suggest that future studies of FGF21 physiology and regulation in humans should consider sex differences in FGF21–fructose dynamics and that metabolic risk may be different based on biological sex.

## 6 Conflict of Interest Statement

None declared.

## 7 Author contributions

M. R., B. H. and J. D. were involved in the study design, study conduct, data analysis and manuscript preparation.
